# Sexual Self-Concept in Women with Disorders/Differences of Sex Development

**DOI:** 10.1007/s10508-021-02188-1

**Published:** 2022-04-01

**Authors:** Nita G. M. de Neve-Enthoven, Nina Callens, Maaike van Kuyk, Chris M. Verhaak, Jan van der Ende, Stenvert L. S. Drop, Peggy T. Cohen-Kettenis, Arianne B. Dessens, on behalf of the Dutch Study Group on DSD

**Affiliations:** 1 Department of Child and Adolescent Psychiatry and Psychology, Erasmus Medical Center Rotterdam-Sophia Children’s Hospital, Sh-1058, P.O. Box 2060, 3000 CB Rotterdam, The Netherlands; 2 Department of Pediatric Endocrinology, University Hospital Ghent and Ghent University, Ghent, Belgium; 3 Department of Medical Psychology, Radboud University Medical Center-Amalia Children’s Hospital Nijmegen, Nijmegen, The Netherlands; 4 Department of Pediatrics, Division of Pediatric Endocrinology, Erasmus MC-Sophia Rotterdam, Rotterdam, The Netherlands; 5 Department of Medical Psychology and Center of Expertise on Gender Dysphoria Amsterdam, UMC-Free University Medical Center Amsterdam, Amsterdam, The Netherlands; 6 Faculty of Medicine and Health Sciences, Department of Internal Medicine and Pediatrics, University Ghent, Ghent, Belgium

**Keywords:** Body image, Difference/disorder of sex development, Female sexual distress and dysfunction, Intersex, Sexual self-schema

## Abstract

Many women born with disorders or differences of sex development (DSD) report sexual problems, in particular women who have undergone extensive genital reconstruction. Examining cognitions and emotions that hinder or promote sexuality may facilitate understanding these sexual problems and may contribute to the development of specific interventions. In this study, sexual self-concept, body image, and sexual functioning were investigated in relation to genital surgery. To conduct the study, the women’s Sexual Self-Concept Scale was translated to Dutch. Evaluation of psychometric properties was conducted in a sample of healthy Belgian and Dutch women participating in an anonymous web-based survey (*N* = 589, Mdn age, 23 years). The resulting three-factor structure corresponded largely to that of the original version. Compared to control women, women born with a DSD who were included in the Dutch DSD study (*N* = 99, Mdn age, 26 years) described themselves as being less interested in sex and less sexually active. These women also harbored more negative emotions and cognitions regarding their sexuality and were less satisfied with their external genitalia. In women with a DSD, sexual self-concept was associated with compromised outcomes on sexual functioning and distress. Women who were in a steady relationship, and/or had been sexually active in the past 4 weeks had a more positive sexual self-concept, took a more active role in their sexual relationship, experienced more sexual desire and arousal and less sexual distress than women who were not involved in a partner relationship. Findings in this study indicate that cognitions and emotions related to sexual self-concept play a role in sexual functioning of women with a DSD. A cognitive behavioral counseling approach with focus on coping and exploration of their own sexual needs could prove useful in this group.

**Supplementary Information:**

The online version contains supplementary material available at 10.1007/s10508-021-02188-1.

## Introduction

Disorders or differences of sex development (DSD) comprise a heterogeneous group of genetic congenital conditions characterized by an atypical development of the sex organs (Hughes et al., 2006; Lee et al., 2016).

In female-reared persons, feminizing surgery is offered in pursuit of a female appearance of the genital and to enable penile–vaginal intercourse, but is criticized for compromising erotic sensation of the genital area including the clitoris, causing discomfort and pain during intercourse (Crouch & Creighton, 2004; Gastaud et al., 2007; Lesma et al., 2014; Minto et al., 2003a, 2003b; Ogilvie et al., 2006; Schonbucher et al., 2010; Speiser et al., 2010; Stikkelbroeck et al., 2003; Wisniewski et al., 2004). The available follow-up data on sexual functioning after genital surgery are conflicting, due to differences in definition of outcome variables and applied methodology (Harris & Chan, 2019).

The sexual development of adolescent girls with DSD differs from that of their age peers. DSD conditions have devastating implications for fertility and sexual intercourse. Affected adolescents girl and women may experience variable levels of distress, connected to perceived compromised womanhood and fear of being devalued by others (Callens et al., 2014). Girls with DSD tend to be older when they engage in romantic and sexual activities (Frisen et al., 2009; Kleinemeier et al., 2010; van der Zwan et al., 2013b). Negative body image, doubts, and anxiety about their sexual competence probably influence this delay (Frisen et al., 2009; Krege et al., 2000; van de Grift et al., 2018). Adult women report decreased sexual interest, experiences, and activity (Callens et al., 2012a; Dittmann et al., 1990a, 1990b; Kreukels et al., 2019; van der Kamp et al., 1992; Zucker et al., 2004). While surgery of the atypical genitalia may compromise erotic sensation and may induce discomfort and pain during sexual activities, psychological factors may play a role in the comprised sexual functioning as well. Genital surgery or vaginal dilation therapy may correct atypical appearance and facilitate sexual intercourse and at the same time underline that she is not a “normal” woman. Such interventions may result in self-disapproval, ideas that her own body is not fit for sexual intercourse and that one cannot be a highly valued partner (Dreger, 1998).

Sexual dysfunctions overall are common in the general population (Kedde, 2012; Laumann et al., 2005). Psychological and interpersonal factors usually play a dominant role in the cause or reinforcement of sexual dysfunctions (Basson et al., 2000; Leiblum & Wiegel, 2002; Middleton et al., 2008). There is a vast body of research examining the role of cognitive factors in hindering or promoting sexual adjustment to a life with a chronic condition, such as self-esteem, coping strategies, and beliefs regarding illness and body image (Middleton et al., 2008). The sexual self-concept is a multidimensional psychological construct that incorporates personal cognitions and emotions about one’s sexuality, which is assumed to influence sexual functioning. Applied to sexuality, the sexual self-concept incorporates cognitive generalizations about sexual aspects of the self that derive from past experiences, affect current experiences, facilitate the processing of sexual information, and guide sexual behavior (Andersen & Cyranowski, 1994). When positive, sexual self-concepts promote positive attitudes and cognitions toward sexuality. Positive sexual self-concepts will facilitate sexual responding and will result in positive experiences, higher frequencies of sexual behavior, low levels of negative sexual affect, and, with regard to relationships, feelings of passionate love and secure romantic attachments. Negative self-concepts facilitate negative attitudes regarding sexuality, which may enhance vulnerability for sexual distress, difficulty, or dysfunction (Kilimnik et al., 2018). In sex therapy, sexual self-concept and related cognitions will be explored to raise awareness of the adverse influence of these negative, dysfunctional cognitions on sexuality (Frühauf et al., 2013). The atypical genital appearance of a woman with DSD may interact with the sexual self-concept and consequently impair sexual functioning, particularly in case of shame about the atypical genital or fear for pain during intercourse. In such cases, romance and sexual relationships might be stressful: the beloved one needs to be informed, the atypical genitalia will be exposed, some adjustments may be required to enable sexual intercourse, there may be fear of rejection, etc. Sex therapy will in such cases focus on the exploration of the sexual self-concept and adaptation of dysfunctional cognitions into more helpful (often more realistic and positive) ones (Frühauf et al., 2013).

Sexual self-concept in relation to compromised sexual and emotional functioning has been studied in women after sexual trauma (Kilimnik et al., 2018; Meston et al., 2006; Reissing et al., 2003), in women with dyspareunia (Pazmany et al., 2013), and in postmenopausal women (Heidari et al., 2017). Findings revealed a less positive sexual self-concept after sexual abuse and associations between negative cognitions related to sexual self-concept in women who reported vaginal pain, sexual discomfort, and distress.

Examination of the role of cognitions in sexuality in DSD can be helpful to understand underlying constructs of sexual problems, but so far received little attention. This study explores genital atypicality on sexual self-concept and the relationship with sexual functioning and body image in women with 46,XX and 46,XY DSD. It was hypothesized they have a more negative sexual self-concept and body image than women without DSD.

Since no Dutch instrument to measure sexual self-concept was available, we have translated the Women’s Sexual Self-Concept Scale (WSSCS) developed by Johnson Vickberg and Deaux (2005) and examined its psychometric properties (Study 1). The WSSCS was selected based on its multifaceted view of women’s sexual self-concept, including descriptive adjectives as well as cognitions and emotions and is relatively balanced in terms of positive and negative aspects of women’s sexuality (Johnson Vickberg & Deaux, 2005). Findings on sexual self-concept in relation to sexual functioning, genital surgery, and body image for women with DSD are described in Study 2.

## Study 1: Psychometric Evaluation of the Dutch Women’s Sexual Self-Concept Scale

### Method

#### Participants

A total of 589 healthy women, aged 18–68 years, participated in an anonymous web-based survey among Dutch-speaking Belgian and Dutch women. The study website provided information on the study purpose and inclusion criteria. Before entering the survey, potential participants digitally gave their informed consent and filled out their gender, age, and health status. For potential participants who did not meet the inclusion criteria, the survey automatically discontinued. All remaining responders were included and continued the survey.

#### Procedure

Potential responders were invited by social media advertisements and by local flyers and posters (Ghent and Rotterdam), to visit a secured study website and fill out the questionnaire. The stated objective in these advertisements was collaboration in a study for the development of a Dutch questionnaire on sexual self-concept for healthy women of 18 years and older. For detailed information of the study, potential responders were referred to the website. Each potential participant who entered the web-based survey was first informed about the study purpose and inclusion criteria for applicants. Each potential responder had to digitally declare that she had been informed about and comprehended the study purpose and give her informed consent before she could continue. Next, in order to assess whether potential participants met the inclusion criteria, participants had to fill out their gender, age, and health status. Health status was established by asking respondents whether they suffered from a neurological or neuromuscular disease, bone and joint disorder, cardiovascular, pulmonary, endocrine, metabolic, gynecological or urinary disease. Potential participants who did not meet the inclusion criteria received a message that were sorry to inform them that they were not eligible for the study. All included responders continued and filled out demographic characteristics and the WSSCS-D (Dutch version).

#### Measures

##### Biographic Questionnaire

Demographic data including age, ancestry, education, and relationship status are listed in Table 1.

**Table 1:** Demographic characteristics of participants in DSD and control groups

	Control	DSD	*p*
*N*	589	99	
*Median age (years, range)*	23 (18–65)	26 (17–60)	**.005**
*Ancestry (%)*			**.030**
Caucasian	96.6	82.8	
Non-Caucasian	2.2	4.0	
Mixed ancestry	1.2	4.0	
Not specified	–	9.1	
*Education (%)*			**.001**
Primary school-lower secondary	1.9	9.1	
Higher secondary school	24.3	62.6	
Higher education short type	18.2	18.2	
Higher education long type-university	54.2	10.1	
Not indicated	1.5	–	
*Partner (% yes)*	78.9	59.6	**< .001**
*Sexual orientation (%)*			**< .001**
Androphylic	88.1	73.7 (total)	NS^a^
46,XY FG		78.8	
46,XY AG		57.7	
46,XX CAH		80.0	
Gynephilic	2.7	9.1 (total)	NS^a^
46,XY FG		9.1	
46,XY AG		15.4	
46,XX CAH		5.0	
Bisexual	1.4	5.1 (total)	NS^a^
46,XY FG		3.0	
46,XY AG		3.8	
46,XX CAH		7.5	
Unsure/unknown	7.8	12.1 (total)	NS^a^
46,XY FG		9.1	
46,XY AG		23.1	
46,XX CAH		7.5	
*Sexually active in the past 4 weeks (% yes)*	94.2	66.7 (total)	**.001**
46,XY FG		54.5	
46,XY AG		80.8	
46,XX CAH		70.0	

Bold values indicate significant values at level *p* < .05

*46,XY FG* 46,XY females born with female typical genital, *46,XY AG* 46,XY females born with atypical female genital, *46,XX CAH* 46,XX females born with atypical female genital (Callens et al., 2016), *NS* not significant

^a^No between-group differences in sexual orientation were found for women with DSD

##### Women’s Sexual Self-Concept Scale (WSSCS)

The self-report WSSCS measures sexual self-concept (Johnson Vickberg & Deaux, 2005) on three scales that reflect women’s attention to and active role in sexuality (Agentic Sexuality), sexual coercion, negative emotions, and concern about impressions (Negative Associations), and responsibility, carefulness, and faithfulness (Reserved Approach). Items were answered on a 7-point Likert scale. Higher scores reflect a more frequent report of the cognitions, emotions, and behaviors included in the scale. The original WSSCS was translated into Dutch and translated back from Dutch to English to enhance translation quality.

#### Data Analysis

To test factors proposed by the original instrument that could be replicated in our sample, a confirmatory factor analyses (CFAs) in latent variable modeling program Mplus was conducted (Muthén & Muthén, 1998–2012). Chi-square test (*χ*^2^, non-significant), chi-square divided by the degrees of freedom in the model (*χ*^2^/*df*, < 2), root-mean-square error of approximation (RMSEA, < .05), comparative fit index (CFI, > .90), and standardized root-mean-square residual (SRMR, ≤ .08) were applied to test for goodness of fit (Muthén & Muthén, 1998–2012). The three-factor structure of the original WSSCS was not replicated in our sample. Excluding items and re-executing CFAs improved goodness-of-fit indices, but most final values were insufficient to meet goodness-of-fit criteria: *χ*^2^ = *p* < .001, *χ*^2^/*df* = 5.39, RMSEA = .09, CFI = .84, except for the SRMR value that had the sufficient value of ≤ .08.

As the results in the CFAs were not acceptable and our data thus did not fit the original model hypothesized, an exploratory principal component analysis (PCA) with the varimax rotation method was applied in IBM SPSS Statistics to identify underlying factors in the Dutch version of the WSSCS-D (IBM Corp., 2016). Factors with eigenvalues > 1 and items with factor loadings > .30 were considered acceptable. Internal consistency estimates were calculated using Cronbach’s *α*.

### Results

#### Biographic Questionnaire

Age, ancestry, education, partnership, sexual orientation, and sexual activity in the past 4 weeks of participants in the DSD and control groups are summarized in Table 1.

#### Psychometric Evaluation of the Dutch Women’s Sexual Self-Concept Scale

The PCA yielded a three-factor structure that corresponded largely to that of the original WSSCS. The constructs measured by factors 1 and 2 had major substantive agreement with the original Agentic Sexuality and Negative Associations scales, and labels were retained. The third factor measured a different construct and was relabeled ‘Loyalty’, since the factor contained items on romantic love and loyalty to a partner in our sample. Internal consistency estimates (*α*’s) of the Dutch Agentic Sexuality and Negative Association subscales were, respectively, excellent and good and that of the Loyalty subscale was relatively low. The modified Dutch version of the WSSCS was named WSSCS-D. Table 2 shows details.

**Table 2:** Exploratory principal components analysis of the Women’s Sexual Self-Concept Scale-Dutch

Item	Factor loadings		
	Agentic sexuality	Negative associations	Loyalty
34. Sensual	**.79**		.18
14. Passionate	**.76**		.21
33. Likely to desire sex	**.73**		
3. Erotic	**.67**	− .10	
39. Aware of own sexual feelings	**.66**	− .22	.15
21. Seductive	**.63**		.16
30. Open about sexuality	**.60**	− .25	
25. Likely to fantasize about sex	**.59**	.13	− .17
8. Likely to experiment	**.56**		
36. Likely to initiate sex	**.55**	− .25	
6. Likely to enjoy sex	**.52**	− .40	.23
22. Expresses sexuality through appearance	**.51**	− .26	
28. Sensitive to partner's needs	**.48**	− .11	.47
29. Insists on having own needs met	**.42**	− .25	
18. Insensitive about partner's needs^a^	.29	.22	− .22
26. Willing to have sex before marriage^a^	.15		
23. Afraid during sex	− .12	**.68**	
24. Likely to feel guilty after having sex		**.64**	
9. Worries about making a good sexual impression on others	− .11	**.61**	
15. Likely to deny feelings of desire	− .13	**.59**	− .19
2. Likely to be anxious about having sex	− .28	**.57**	
11. Likely to pretend to enjoy sex	− .16	**.53**	− .14
32. Careful about sex^b^	− .17	**.53**	.15
13. Concerned about getting a bad sexual reputation		**.53**	− .18
12. Passive about voicing own sexual desires	− .24	**.51**	− .16
20. Likely to be taken advantage of		**.51**	− .12
7. Likely to be depressed after having sex	− .14	**.50**	− .10
4. Sexually repressed	− .12	**.50**	
16. Feel pressured to have sex	− .25	**.47**	− .15
10. Forced to have sex		**.42**	− .13
37. Confused about sexuality		**.42**	− .18
17. Knowledgeable about own body^c^		.40	.14
5. Let partner take initiative in sex	− .28	**.37**	
19. Confused about sexual orientation		**.31**	− .17
1. In a relationship^d^	− .19		**.52**
35. Faithful to a partner		− .14	**.51**
27. In love^d^	.32	− .15	**.46**
38. Romantic^d^	.31		**.46**
31. Responsible for protection from STD's^a^			.27
*Cronbach's alpha*	.90	.86	.56

Based on data obtained in the web-based survey (*n* = 589)

Bold values indicate item loading on a specific factor

^a^Exclusion from the instrument

^b^Item was included in Factor 3 on the original scale

^c^Content did not correspond with original item, excluded

^d^Items were included in Factor 1 on the original scale

## Study 2: Sexual Self-Concept in Women with DSD

### Method

#### Participants

The DSD group consisted of 99 adult Dutch women aged 17–60, who consulted the DSD Teams in Erasmus Medical Center Rotterdam (*n* = 54), Radboud University Medical Center Nijmegen (*n* = 32), and the VU Medical Center Amsterdam (*n* = 13) between 2007 and 2012 (Callens et al., 2012a, 2012b, 2016; de Neve-Enthoven et al., 2016; van der Zwan et al., 2013a, 2013b).

Only women with a molecularly or biochemically confirmed diagnosis were included. Women with (1) additional congenital conditions, (2) cloacal/bladder malformations, (3) Turner syndrome, and (4) Mayer–Rokitansky–Küster–Hauser syndrome were excluded from the study. The control group comprised 589 women from Study 1.

#### Procedure

Initially, 172 patients were identified who met inclusion criteria. All were invited to participate in a follow-up audit; 99 women (58%) joined the study (Callens et al., 2012a, 2012b, 2016; de Neve-Enthoven et al., 2016; van der Zwan et al., 2013a, 2013b).

To examine the influence of genital atypicality on sexual self-concept and the relationship with sexuality outcomes in women with DSD, participants were divided into three subgroups according to karyotype and degree of genital virilization at birth, as this reflects prenatal androgen action and is related to number and type of postnatal genital surgeries: (1) 33 women with a 46,XY karyotype and a female appearance of the genitalia, (2) 26 women with a 46,XY karyotype born with hypovirilization of the genitalia, and (3) 40 women with a 46,XX karyotype born with various degrees of virilization of the genitalia (de Neve-Enthoven et al., 2016). See Table 3 for relevant medical information.

**Table 3:** Diagnosis information and details of the medical treatments within the DSD group

	46,XY females born with female appearance of the genital (*n* = 33)	46,XY females born with hypomasculinization of the genital (*n* = 26)	46,XX females born with masculinization of the genital (*n* = 40)
Diagnosis (*n*)	CAIS (21)	Partial GD (7)	CAH CYP21A (38)
	Complete GD (12)	17β HSD (8)	CYP11B1 (2)
		PAIS (5)	
		Leydig cell hypoplasia (3)	
		NR5A-1 (1)	
		17,20 LD (1)	
		Hypomasculinization, unknown cause (1)	
Genital surgery (%, *n*)	27.3% (9)	80.8% (21)	90% (36)
Type of genital surgery (*n*)	Introitoplasty (2)	Vaginoplasty, introitoplasty and clitoroplasty (5)	Vaginoplasty, introitoplasty and clitoroplasty (9)
	Vaginoplasty and clitoroplasty (4)^a^	Vaginoplasty (3)	Introitoplasty (1)
	Vaginoplasty (3)^b^	Clitoroplasty (3)	Clitoroplasty (4)
		Vaginoplasty and introitoplasty (3)	Vaginoplasty and clitoroplasty (14)
		Vaginoplasty and clitoroplasty (5)	Introitoplasty and clitoroplasty (8)
		Introitoplasty and clitoroplasty (2)	
Median number of surgeries (range)	0 (0–2)	1 (0–4)	2 (0–4)
Median age of first surgery (range)	16 (0–28)^c^	16 (0–27)	3 (0–19)^d^
Vaginal dilation therapy only (%, *n*)	21.2% (7)^b^	7.7% (2)	0% (0)
No genital treatment (%, *n*)	51.5% (17)	11.5% (3)	10% (4)
Gonadectomy (%, *n*)	93.9% (31)	100% (26)	–

All patients with DSD were under medical supervision at Erasmus Medical Center Rotterdam (*n* = 54), Radboud University Nijmegen Medical Center (*n* = 32), and the VU Medical Center Amsterdam (*n* = 13)

*17β HSD* 17β hydroxysteroid dehydrogenase deficiency type 3, *17, 20 LD* 17,20 lyase deficiency, *CAH* congenital adrenal hyperplasia, *CAIS* complete androgen insensitivity syndrome, *CYP21A* 21-hydroxylase deficiency, *CYP11B1* 11β-hydroxylase deficiency, *GD* gonadal dysgenesis, *NR5A-1* NR5A-1 gene mutation, *PAIS* partial androgen insensitivity syndrome, *46 XY* karyotype with hypomasculinization of unknown cause, despite extensive analysis

^a^All complete GD

^b^All CAIS

^c^ Median (range) age of first surgery: CAIS 20 (16–28), complete GD: 3 (0–19), *p* = .035

^d^This group comprised one woman with late onset CAH

Participants were asked to fill out all questionnaires, but were free to turn down questionnaires or individual items. Tables 4, 5, and 6 display the numbers of completed rating scales.

**Table 4:** Median scores and range of scores on the Women’s Sexual Self-Concept Scale-Dutch

	Agentic sexuality Mdn (range)	Negative associations Mdn (range)	Loyalty Mdn (range)
DSD (*n* = 87)	65 (14–98)	35 (16–81)	23 (6–28)
Control (*n* = 589)	73 (17–98)	28 (16–85)	22 (4–28)
*p* value	**< .001 phasis>**	**.014**	.255
46,XY female typical genital (*n* = 28)	66.5 (16–91)	32 (16–79)	22 (6–28)
46,XY atypical genital (*n* = 21)	60 (15–98)	36 (18–64)	24 (6–28)
46,XX atypical genital (*n* = 38)	68 (14–90)	31 (17–81)	23 (10–28)
*p* value	.348	.896	.750
DSD, steady relationship (*n* = 54)	66 (27–98)	33 (17–72)	24.5 (12–28)
DSD, no steady relationship (*n* = 33)	65 (14–91)	36 (16–81)	21 (6–28)
*p* value	.933	.801	**.001**
DSD, sexually active (*n* = 59)	67 (27–98)	35 (17–81)	24 (15–28)
DSD, not sexually active (*n* = 23)	57 (14–91)	28 (16–68)	18 (6–27)
*p* value	**.013**	.466	**< .001**

Bold values indicate significant values at level *p* < .05

*Absolute scoring ranges.* Agentic Sexuality 14–98, Negative Associations 16–119, Loyalty 4–28. Higher scores reflect a more frequent report of the cognitions, emotions and behaviors included in the scale

*Mdn* median score, *WSSCS-D* Women's sexual self-concept scale-Dutch

**Table 5:** Mean scores and SDs on the Body Image Scale

	External genitals *M* (SD)	Other sex-specific characteristics *M* (SD)	Non-sex-specific characteristics *M* (SD)
DSD (*n* = 81)	8.48 (2.62)	17.05 (4.04)	52.68 (11.80)
Control (*n* = 79)	6.97 (1.75)	18.10 (3.37)	52.92 (10.34)
*p* value	**< .001**	.229	.196
46,XY female typical genital (*n* = 28)	8.39 (2.44)	17.21 (3.00)	53.43 (8.63)
46,XY atypical genital (*n* = 23)	8.96 (2.36)	16.78 (4.95)	51.00 (13.82)
46,XX atypical genital (*n* = 30)	8.20 (2.99)	17.10 (4.23)	53.27 (12.92)
*p* value	.574	.929	.726
DSD, steady relationship (*n* = 55)	8.47 (2.95)	16.12 (4.00)	52.04 (12.84)
DSD, no steady relationship (*n* = 40)	8.63 (2.24)	18.68 (3.73)	56.80 (11.68)
*p* value	.775	**.004**	.077
DSD, sexually active (*n* = 63)	8.46 (2.83)	16.72 (4.09)	54.09 (13.77)
DSD, not sexually active (*n* = 27)	8.48 (2.19)	18.00 (4.18)	53.74 (10.22)
*p* value	.972	.206	.911

Bold values indicate significant values at level *p* < .05

*Absolute scoring ranges.* External genitals: 3–15, Other sex-specific characteristics: 7–35, Non-sex-specific characteristics: 23–115. Higher scores reflect greater dissatisfaction

*M* mean score, *SD* standard deviation

**Table 6:** Multiple regression analyses testing associations with current sexual functioning and distress

	FSFI desire (*n* = 66) *R*^2^ = .49, *F*(1,40) = 6.27, *p* < .001				FSFI arousal (*n* = 66) *R*^2^ = .39, *F*(1,40) = 4.13, *p* = .002				FSFI lubrication (*n* = 63) *R*^2^ = .32, *F*(1,39) = 3.04, *p* = .02				FSFI orgasm (*n* = 63) *R*^2^ = .25, *F*(1,38) = 2.20, *p* = .06			
	*B* (SE)	95% CI		*p*^a^	*B* (SE)	95% CI		*p*^a^	*B* (SE)	95% CI		*p*^a^	*B* (SE)	95% CI		*p*^a^
		L	U			L	U			L	U			L	U	
Constant	1.31 (1.06)	− .84	3.46	.23	**4.99 (1.39)**	2.19	7.80	< **.0001**	**5.41 (1.79)**	1.79	9.04	< **.001**	5.44 (2.25)	.88	10.00	.02
WSSCS-D: agentic sexuality	**.03 (.01)**	.012	.05	**< .001**	.01 (.01)	− .01	.04	.24	**.05 (.02)**	.02	.08	< **.001**	.03 (.02)	− .01	.07	.11
WSSCS-D: negative associations	− .01 (.01)	− .03	.00	.10	− **.02 (.01)**	− .05	− .00	.03	− .02 (.01)	− .04	.01	.29	− .03 (.02)	− .06	.01	.11
WSSCS-D: loyalty	− .00 (.04)	− .08	.08	.97	− .00 (.05)	− .10	.10	.95	− .12 (.06)	− .25	.01	.07	− .04 (.08)	− .21	.12	.60
BIS: external genitals	.02 (.05)	− .08	.12	.67	− .06 (.07)	− .19	.07	.35	− .01 (.08)	− .18	.16	.91	− .08 (.11)	− .29	.14	.48
Total number of genital surgeries	**.36 (.12)**	.12	.60	< **.001**	**.41 (.16)**	.09	.72	.01	.07 (.20)	− .34	.48	.72	− .25 (.26)	− .77	.28	.35
Satisfaction with genital functionality	.25 (.17)	− .10	.59	.16	− .06 (.22)	− .51	.39	.80	− .20 (.29)	− .78	.39	.50	.02 (.37)	− .74	.78	.96

	FSFI satisfaction (*n* = 46) *R*^2^ = .21, *F*(1,26) = 1.12, *p* < .38				FSFI pain (*n* = 49) *R*^2^ = .24, *F*(1,29) = 1.56, *p* = .20				Global satisfaction (*n* = 84) *R*^2^ = .39, *F*(1,49) = 5.11, *p* = .001				FSDS-R (*n* = 95) *R*^2^ = .41, *F*(1,42) = 5.95, *p* = .001			
	*B* (SE)	95% CI		*p*^a^	*B* (SE)	95% CI		*p*^a^	*B* (SE)	95% CI		*p*^a^	*B* (SE)	95% CI		*p*^a^
		L	U			L	U			L	U			L	U	
Constant	2.57 (2.15)	− 1.84	6.98	.24	1.96 (4.01)	− 6.23	10.16	.63	**3.45 (.96)**	1.52	5.38	**< .001**	8.20 (7.92)	− 7.68	24.09	.31
WSSCS-D: agentic sexuality	.00 (.02)	− .03	.04	.78	− .01 (.03)	− .07	.06	.84	.01 (.01)	− .01	.03	.30	− **.18 (.07)**	− .33	− .03	**.02**
WSSCS-D: negative associations	.00 (.02)	− .03	.04	.88	− .02 (.03)	− .08	.05	.62	− .02 (.01)	− .04	− .00	.02	**.23 (.07)**	.08	.37	< **.001**
WSSCS-D: loyalty	.10 (.09)	− .08	.27	.26	.15 (.14)	− .12	.43	.27	.02 (.03)	− .04	.09	.49	.10 (.27)	− .44	.63	.72
BIS: external genitals	− .06 (.08)	− .23	.11	.49	.08 (.19)	− .31	.47	.69	.06 (.06)	− .06	.18	.29	.17 (.48)	− .79	1.13	.73
Total number of genital surgeries	.29 (.18)	− .08	.67	.12	− .13 (.48)	− 1.11	.85	.79	.25 (.12)	.00	.50	.048	− .98 (1.01)	− 3.00	1.04	.34
Satisfaction with genital functionality	.03 (.31)	− .61	.66	.94	− 1.19 (.70)	− 2.63	.25	.10	− .53 (.20)	− .93	− .12	.01	2.13 (1.66)	− .21	5.46	.21

*R*^2^ = percent of total variation that can be explained by the model. *F* tests the overall fit of the model. *F*(*df*1, *df*2) = (degrees of freedom 1, degrees of freedom 2)

*FSFI* Female sexual function index, *FSDS-R* female sexual distress scale-revised, *WSSCS-D* Women's Sexual Self-Concept Scale-Dutch, *BIS* body image scale, *B* = unstandardized regression coefficient, strength of relationship between predictor and outcome in the units of measurement of the predictor. *SE* standard error of B, *95% CI* = 95% confidence interval. Range of values around B believed to contain, with a 95% probability, the true value of B. *L* lower value, *U* upper value

^a^Adjustment for multiple comparisons: Bonferroni. Significance level *p* < .007

#### Measures

##### Biographic Questionnaire and the Women’s Sexual Self-Concept Scale-Dutch Version

See Study 1.

##### Medical Data

Medical data with respect to diagnosis, genital virilization at birth, genital surgery, and dilation therapy were retrieved from medical files. Genital phenotype at birth was described according to Sinnecker (Sinnecker et al., 1996) or by clitoral hypertrophy and the level of confluence of the vagina and urethra in women with 46,XX DSD (van der Zwan et al., 2013b).

#### Sexuality Parameters

*Sexual orientation* was assessed by the self-identification item of the Klein Sexual Orientation Grid (Dessens et al., 1999; Klein, 1978).

*Sexual activity status* indicates whether or not women had been sexually active in the 4 weeks prior to self-evaluation.

*Sexual functioning* was assessed with the Female Sexual Function Index (FSFI; Rosen et al., 2000; ter Kuile et al., 2006). Only 24% of women with DSD had a partner and had had sexual intercourse with vaginal penetration in the past 4 weeks. As the total FSFI score could only be calculated for 24 women, this score was excluded from further analyses (Callens et al., 2012a, 2012b; van der Zwan et al., 2013b). The FSFI-subscales were completed by representative numbers of respondents; statistical analyses were performed with the FSFI-subscales and Item 16, which measures global sexual satisfaction.

*Sexual distress* was assessed by the Female Sexual Distress Scale-Revised (FSDS-R; Derogatis et al., 2008; ter Kuile et al., 2006)).

*(Dis)satisfaction with the functionality of the genitalia* was assessed on a 3-point Likert-scale ranging from *1* (dissatisfied) to 3 (satisfied).

*Body image* was measured by an adapted version of the Body Image Scale (BIS; Baardman & De Jong, 1984; Lindgren & Pauly, 1975; Secord & Jourard, 1953)). All body parts were rated on a five-point Likert scale, ranging from 1 (very satisfied) to 5 (very dissatisfied). Three scales were composed: (1) (dis)satisfaction with the external genital (3 items: vagina, clitoris, labia); *α* = .91), (2) (dis)satisfaction with non-genital sex-specific body parts (7 items, e.g., breasts, hips); *α* = .64), and (3) (dis)satisfaction with non-sex-specific body parts (23 items, e.g., nose, eyes, hair); *α* = .91). Reference data for the BIS were provided by 79 Dutch female students who participated for extra credit in an undergraduate psychology course (aged 18–43, *M* = 21.6 years, SD = 3.5).

#### Data Analysis

Chi-square or Fisher’s exact tests were used to compare categorical parameters and domains scores between independent groups. Analysis of variance (ANOVA) was used to compare differences in continuous variables with normal distribution between two or more groups. Internal consistency estimates were calculated using Cronbach’s *α*. In case of skewed distributions, the nonparametric Mann–Whitney *U* (MW) test was used to compare differences in continuous variables with skewed distributions between two independent groups. The nonparametric Kruskal–Wallis (KW) tests were used to compare continuous variables with skewed distributions among three or more independent groups. Findings on significant KW tests were followed up by post hoc MW testing, comparing pairs of group medians, followed by Bonferroni corrections to adjust for multiple testing. Associations between sexual self-concept (WSSCS-D) and sexuality outcomes (FSFI, FSDS-R) in women with DSD were assessed with multiple linear regression analyses, followed by Bonferroni corrections to adjust for multiple testing for the FSFI scales. Dependent variables were the FSFI domain scores, FSFI Item 16, and the FSDS-R total score. Independent variables included all three WSSCS subscales, the BIS external genital scale, total number of surgeries, and satisfaction with genital functionality. Two-tailed statistical tests were chosen to reduce the risk of type *I* errors. *p* values < .05 or adjusted *p* values in case of Bonferroni correction were considered statistically significant. All analyses were executed in IBM SPSS Statistics (IBM Corp, 2016).

### Results

#### Participant Characteristics

**Fig. 1:**
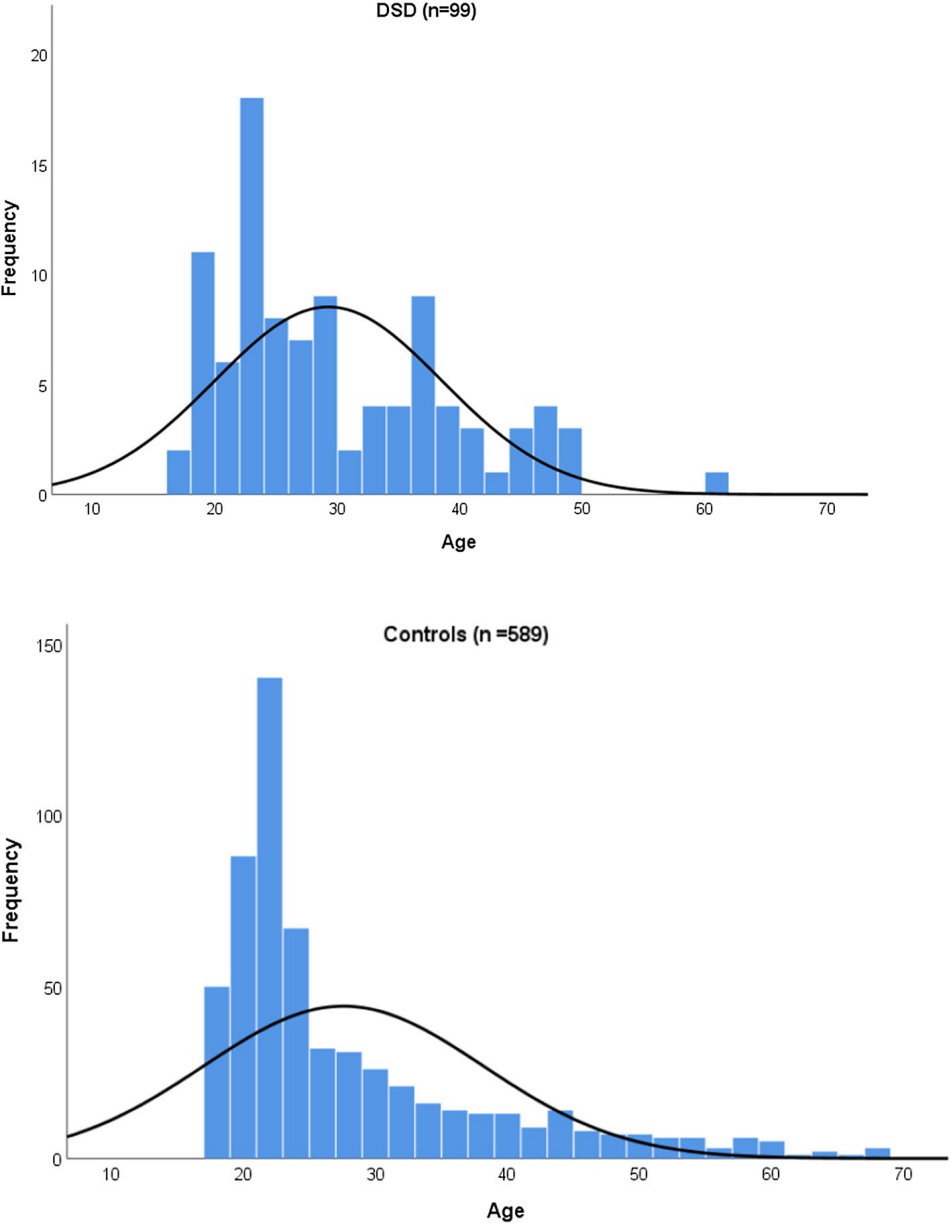
Representation of age in DSD and control groups

Table 1 summarizes characteristics of DSD and control groups. Women in the DSD group were slightly older than controls. Although women up to 60 years of age were invited to take part, the majority of participants in both groups were aged 20–40, see age represented in Fig. 1. The figure reveals that particularly in the control group, age was skewed.

Compared to controls, more women with DSD were born to consanguine parents. On average, women in the control group were highly educated and more than half of control women reached the university level. However, the educational level of women with DSD is representative for the entire Dutch-speaking population.

Women with DSD were also less involved in romantic relationships and had been less sexually active in the past 4 weeks. As reported in previous studies (Callens et al., 2016; Meyer-Bahlburg et al., 2008), women with 46,XY and 46,XX born with atypical genitalia more often reported a bisexual or gynephilic orientation than control women.

#### Sexual Self-Concept

On the WSSCS-D Agentic Sexuality and Negative Association scales, women with DSD reported to be less interested in sex and less sexually active and had more negative associations concerning themselves as a sexual person than control women. There were no between-group differences on the Loyalty scale.

Genital virilization at birth was not associated with sexual self-concept; women from all DSD subgroups responded similarly on all WSSCS-D scales. However, women with DSD who were involved in a steady relationship valued romance and loyalty to a partner more highly (Loyalty, *p* < .001) than women with DSD who were not involved in a steady relationship. Women with DSD who had been sexually active in the past 4 weeks reported to take a more active role in sex (Agentic Sexuality *p* = .013) and also reported to value loyalty more than women who had not been sexually active (Loyalty, *p* < .001). For details, see Table 4.

Subgroup analyses were conducted to explore the effect of age, consanguinity, and educational level on the WSSCS-D. The influence of age as a confounding factor was reduced by homogenization of DSD and control groups; analyses were performed again in women aged 20–40. No influence of age was found; differences between DSD and control women regarding evaluation of their sexual self-concept were repeated (see Supplementary Table 1). The DSD group comprised more women of non- and mixed Caucasian ancestry and individuals who had not answered the question on ancestral background. Could findings between DSD and control groups on sexual self-concept (partially) be attributed to differences between groups in ancestry? To answer this question, analyses were repeated for the subgroups of Caucasian subjects with DSD and controls only. Findings in Supplementary Table 2 showed that inclusion of individuals of non- and mixed Caucasian ancestry and individuals who had not filled out their ancestry (*n* = 13) hardly affected findings on the WSSCS-D. Finally, additional subgroup analysis with educational level as independent and WSSCS-D as dependent variable demonstrated that different educational levels did not differ on sexual self-concept outcomes (Supplementary Table 3).

#### Body Image

In general, women with DSD reported significantly more dissatisfaction with their external genitalia than control women (*p* < .001), but equal satisfaction with respect to non-genital sex-specific body parts and non-sex-specific body parts (Table 5).

Degree of genital virilization at birth and body satisfaction were not related: Women from all DSD subgroups were equally satisfied with their body. Women with DSD who were in a steady relationship were more satisfied with the appearance of their other-sex-specific characteristics than women with DSD who did not have a partner (*p* = .004), but no differences were found regarding non-sex-specific characteristics. Women with DSD who had been sexually active in the past 4 weeks and women who were not reported equal body satisfaction.

For women with DSD, a significant association was found between the negative cognitions reported on the WSSCS-D Negative Associations scale and degree of dissatisfaction with the external genital (*r* = .409, *p* < .001).

The effect of age, consanguinity, and educational level on body image was assessed too. Subgroup analysis in women aged 20–40 was highly corresponded with findings in the entire DSD and control groups (see Supplementary Table 4). Also regarding ancestry subgroup analysis in Caucasian women only, no differences were found compared to analysis in the entire groups of DSD and control women (Supplementary Table 5). Different educational levels did not reveal different outcomes on the Body Image Scale (Supplementary Table 6).

#### Sexual Self-Concept as Predictor of Sexuality Outcomes for Women with DSD

Table 6 summarizes the results of multiple linear regression analyses. Significant models accounted for 21–49% of variance in the outcome variables. Women who reported to be more sexually active and to take greater sexual initiative (higher scores on Agentic Sexuality scale) reported more sexual desire, less problems with lubrication (FSFI Desire and Lubrication scales), and less sexual distress (FSDS-R). Women who reported more negative sexual associations (higher scores on Negative Associations scale) reported more sexual distress (FSDS-R). The Loyalty scale correlated significantly with the Agentic Sexuality scale (*r* = .65, *p* < .01) and did therefore not individually predict sexuality outcomes. Emotions and behaviors indicative of romantic, loving, and intimate attachments seem to be of significant importance to women with a positive sexual self-concept.

More frequent genital surgery was related to higher scores on sexual desire (FSFI Desire scale). Sexual self-concept, (dis)satisfaction with the external genitalia, (dis)satisfaction with genital function or number of genital surgeries were not associated with the FSFI Arousal, Orgasm, Satisfaction, and Pain scales and global sexual satisfaction (FSFI Item 16).

## Discussion

Women with DSD meet many challenges in romance and sexuality. This study on the relationship between cognitions and emotions and sexuality outcomes revealed that women with DSD had a more negative sexual self-concept than women without DSD (Mueller et al., 2016). Women with DSD described themselves as being less interested in sex and less sexually active than control women did. They reported more negative emotions and cognitions related to sexuality, but did not differ from non-affected women regarding valuing romance and loyalty.

Negative sexual cognitions and emotions were associated with increased sexual distress. Women with DSD who were in a steady relationship and/or had been sexually active in the past 4 weeks had a more positive sexual self-concept, took a more active role in their sexual relationship and highly valued loyalty to a partner. Women with DSD who took a more active role in sexuality experienced higher levels of sexual desire and reported better lubrication and less sexual distress. These findings are in line with findings in previous studies in women with CAH (Zucker et al., 2004).

Women with DSD were reported to be less satisfied with their external genitalia than controls. Dissatisfaction with the appearance of their external genitalia was associated with more negative emotions and cognitions toward sexuality.

Women who had undergone more genital surgeries reported higher levels of sexual desire. We did not observe a relationship between genital surgery and other factors of sexual functioning or sexual distress.

Not being involved in a partner relationship and reduced sexual activity are common among women with DSD (Callens et al., 2012b; Dittmann et al, 1992; Frisen et al., 2009; Gastaud et al., 2007; Köhler et al., 2012; Krege et al., 2000; Kühnle et al., 1995; Lesma et al., 2014; Minto et al., 2003a, 2003b; Nordenström et al., 2010; Schonbucher et al., 2010; Stikkelbroeck et al., 2003; van der Zwan et al., 2013b; Zucker et al., 2004). A relationship with genital surgery and compromised sexual functioning has been observed in women who had the most invasive and multiple reconstructions (Minto et al., 2003b; Nordenström et al., 2010; van der Zwan et al., 2013b). Diminished sexual desire is also observed among women with CAIS and MRKH, who are born with a female vulva and never underwent genital surgery but obtained vaginal length by vaginal dilation (Both et al., 2018; Callens et al., 2012a, 2012b, 2014). Diminished sexual interest in DSD can be explained by several factors such as psychological inhibitions and diminished erotic sensitivity due to reduced vascularization and innervation of the neovagina, and iatrogenic effects of hormonal therapy (e.g., over-suppression of androgens by high glucocorticoid replacement in CAH) (Both et al., 2018; van der Zwan et al., 2013b).

A positive sexual self-concept may instigate a more agentic attitude toward sexuality, but the opposite, a more agentic attitude will instigate sexual involvement resulting in more positive sexual experiences including a positive sexual self-concept, may also be true. Alternatively, women who are not or seldom sexually active may lack sufficient experiences to acquire a more positive sexual self-concept.

Findings in this study indicate that lack of sexual interest and initiatives, negative emotions, and cognitions toward sexuality can be associated with diminished sexual activity. Sex therapy, focused on awareness of the impeding effects of negative cognitions and emotions and adaptation of such cognitions and emotions into more positive ones, may improve the women's sex life (Leiblum & Wiegel, 2002; Middleton et al., 2008; Mueller et al., 2016). Studies on sexuality in women with DSD have mainly tried to identify causes of sexual dysfunctions. These studies demonstrated the complexity of all interacting biological, medical, psychological, and social factors involved. Less effort has been made to develop and investigate DSD-specific interventions aimed to reinforce acceptance and coping with one’s atypical genitalia, exploration, and fulfillment of one’s sexual needs and other types of therapies that can be successful in conquering sexual complaints of individuals with DSD. Application of interventions that may improve sexual quality of life has already been developed but should be adapted for women with DSD. At present, it seems appropriate to further develop and systematically evaluate such interventions to prove their therapeutic value.

A strength of this study is the large group of women with DSD with detailed information on diagnosis, medical and surgical interventions. The psychometric properties of the Dutch version of the main instrument, the WSSCS-D, was evaluated in a large group of control women.

This study has several limitations. First, the design of the study. Study 2 is an unmatched retrospective cohort study of cases (women with DSD) and control women (female psychology students and women recruited by flyers and social media advertisement). Such a design misses the preferred methodological rigor. Any differences between patient and control groups should therefore be considered uncertain. The study requires replication using a more rigorous recruitment strategy.

A second limitation is the patient group. DSD is an umbrella term that comprises a variety of diagnoses with a variety of anatomical and biochemical pathways leading to genital ambiguity, resulting in a great heterogeneity between patients in genital and sexual functioning, even within patient subgroups. DSD conditions are rare or extremely rare. Outcome variables that measure subjective experiences, such as measures on psychosexual functioning, have a large variability. In studying psychosexuality in women with DSD, the researcher who wants to conduct a quantitative study has to group women with different types of DSD diagnoses, being aware that grouping introduces a great heterogeneity in the study group that may affect statistical comparisons. Despite restricted options for statistical analyses, if carefully carried out, this strategy has proven to be informative; we know much more today on the impact of DSD on women’s lives than we did 20 years ago (Callens et al., 2016; de Neve-Enthoven et al., 2016; Jürgensen et al., 2007, 2013; Kleinemeier et al., 2010; Migeon et al., 2002; Minto et al., 2003b; Pappas et al., 2008; Schonbucher et al., 2010; Wisniewski et al., 2004).

The third limitation is the control group. Given the nature of DSD and its impact on patients’ sexual development, it is difficult to compile a proper control group. Women with DSD differ from other women in different areas: they may have more mildly masculinized physical characteristics, may be less feminine in gender behavior and interests, underwent more medical interventions early in life, and many of them need lifelong medical follow-up. From preschool age onward, they have to deal with being slightly different from other girls (Callens et al., 2016).

We initially aimed for a matched control design to control for age and several socioeconomic variables, but this appeared not to be feasible. The number of potential-matched control subjects was limited, and many controls refused participation in the study. So, for measures without normative data we had to search alternative routes to collect control data and opted for students (BIS) and a web-based survey (WSSCS). Recruiting among undergraduate female students, by flyers and social media advertisement, is quickly but may easily generate an unrepresentative skewed sample (Erens et al., 2014). In our study, DSD and control groups significantly differed regarding age, ancestry, and education.

In order to find out whether our findings had been confounded by group differences in age, ancestry, and education, subgroup analyses were executed. Figure 1 shows that the large majority of participating women were aged 20–40. Comparison of women from the DSD and control groups aged 20–40 on sexual self-concept and body image demonstrated again that DSD and control groups differed regarding their evaluation of their sexual self-concept (Supplementary Table 1) and body image (Supplementary Table 4). Findings in this subgroup analyses highly corresponded with findings in the entire DSD and control groups. Although age is a confounding factor in this study, the severity of this confounder is limited.

Compared to the control group, individuals of non-Caucasian and mixed Caucasian ancestry and individuals who had not answered the question on ancestral background were overrepresented in the DSD group. This raised the following question: Can findings between DSD and control groups on sexual self-concept and body image (partially) be attributed to differences between groups in ancestry? To answer this question, analyses were repeated for the subgroup of Caucasian DSD and control subjects only. Findings displayed in Supplementary Tables 2 and 5 show that differences in sexual self-concept and body image between DSD and the control groups were hardly affected by the inclusion of non- and mixed Caucasian subjects and subjects who had not filled out their ancestral background.

In order to study education level as a confounding factor, additional subgroup analyses were conducted with educational level as independent variable on WSSCS and body image as dependent variables. The findings in Supplementary Tables 3 and 6 show that women with different educational levels did not differ on sexual self-concept or body image. However, results should be interpreted with caution, since some of the subgroups are very small. This could have resulted in a lack of power to demonstrate certain differences. From these subgroup analyses, we conclude that although DSD and control groups differed significantly regarding age, ancestry, and educational level, the confounding impact of these variables is limited.

Fourth, about half of the eligible individuals with DSD declined participation, for unspecified reasons. Although the non-responders and participant groups did not differ with respect to diagnosis, medical treatment, attended medical center, age or living in urban or rural areas, it is possible that they differ in other aspects (Callens et al., 2012b, 2016; van der Zwan et al., 2013b).

Fifth, Fig. 1 shows the majority of participants were born after 1980. Their experiences may not be representative for the entire group of Dutch women with DSD. They may have benefitted from the advances in medical knowledge and modern clinical approaches while growing up in a society with an expanding openness and tolerance regarding gender variance and sexuality.

This study focused on sexual self-concept in women with DSD. We initiated a similar study in men with DSD, using the Men’s Sexual Self-Schema (Andersen et al., 1999), but the number of men who agreed to discuss their sexual functioning was small; obtained data therefore could be characterized as anecdotal and have not been statistically analyzed (van der Zwan et al., 2013a).

There are a few studies conducted on sexual dysfunctions in men who had been born with hypospadias and had undergone hypospadias repair-genital reconstruction. Findings indicate that particularly men who needed more extensive genital reconstructions reported sexual dysfunctions (Benson et al., 2018; Migeon et al., 2002; Miller & Grant, 1997; Wilcox & Snodgrass, 2006) but also men with milder types of hypospadias have different comfort levels regarding their sexuality and body image than the overall population of men (Tack et al., 2020). The number of conducted studies that included men who needed extensive reconstructions is small and applied methodology diverse, making it difficult to reach conclusions. Studies that included measures on sexual quality of life in addition to measures on erectile function suggest that the guiding hypothesis in this study may also apply to boys and men with DSD.

Elective surgery for children with genital atypicalities is under debate and a moratorium has been demanded, but recent survey revealed that the majority of patients with DSD approved early genital surgery (Bennecke et al., 2021; Tack et al., 2020). Studies conducted in the past 20 years significantly expanded our knowledge on experienced sexual problems and changed clinical management, as we became aware that genital surgeries may improve appearance and enable coitus but cannot fully restore sexual functioning, particularly not in patients who had been more affected.

In conclusion, sexuality research in patients with DSD has demonstrated the complexity of numerous interacting biological, medical, psychological, and social factors (Wisniewski & Tishelman, 2019). Our study on sexual self-concept in women with DSD revealed that cognitive–emotional factors play a role as well in the experience of sexual problems in these women. Improvement of sexual functioning for women with DSD might be reached by interventions focused on acceptance and coping with the atypical genitalia and exploration and fulfillment of the own sexual needs. A cognitive behavioral counseling approach could prove useful in this group, as cognitive techniques may alter negative cognitions, which are related to the external genital area, and increase positive feelings about one’s sexuality in addition to one’s acquisition of new or different sexual experiences.

## Supplementary Information

Below is the link to the electronic supplementary material.

Supplementary file1 (DOCX 35 KB)

## Data Availability

We do not wish to share data originating from our database. Participants gave their informed consent for this study, but permission has not been obtained to share data widely with other investigators and would require individual consent/assent.
