# Supplementary material for: Sexual Self-Concept in Women with Disorders/Differences of Sex Development
Source: Arch Sex Behav. 2022 Apr 1;51(4):2213–29. doi: 10.1007/s10508-021-02188-1 (PMC9192466; doi:10.1007/s10508-021-02188-1)
Supplement: Supplementary file 1 — Supplementary file1 (DOCX 35 KB) [file 10508_2021_2188_MOESM1_ESM.docx]

**Supplementary tables: Additional analyses on the WSSCS-D and BIS regarding age, ancestry, and educational level**

| **Supplementary Table 1**  *Median scores and range of scores on the WSSCS-D in DSD and control women aged 20-40 years* | | | |
| --- | --- | --- | --- |
|  | Agentic  Sexuality *Mdn (range)* | Negative  Associations *Mdn (range)* | Loyalty *Mdn (range)* |
| DSD (*n* = 66) | 67 (14-98) | 35 (17-81) | 22 (6-28) |
| Control (*n* = 421) | 73 (17-97) | 28 (16-85) | 22 (4-28) |
| *p-value* | ***.012*** | ***.038*** | *.390* |
| *Abbreviations. Mdn =* Median score; WSSCS-D = Women's Sexual Self-concept Scale-Dutch.  *Absolute scoring ranges.* Agentic Sexuality 14-98, Negative Associations 16-119, Loyalty 4-28. Higher scores reflect a more frequent report of the cognitions, emotions and behaviors included in the scale. Higher scores reflect a more frequent report of the cognitions, emotions and behaviors included in the scale. Bold-printed values indicate significant values at level p<.05 | | | |

| **Supplementary Table 2**  *Median scores and range of scores on the WSSCS-D in Caucasian DSD and control women* | | | |
| --- | --- | --- | --- |
|  | Agentic Sexuality *Mdn (range)* | Negative Associations *Mdn (range)* | Loyalty *Mdn (range)* |
| DSD ( *n* = 72) | 63.5 (14-98) | 35.5 (16-81) | 22 (6-28) |
| Control (*n* = 569) | 73 (17-98) | 28 (16-85) | 22 (4-28) |
| *p-value* | ***<.001*** | ***.042*** | *.531* |
| *Abbreviations. Mdn =* Median score; WSSCS-D = Women's Sexual Self-concept Scale-Dutch. | | | |
| *Absolute scoring ranges.* Agentic Sexuality 14-98, Negative Associations 16-119, Loyalty 4-28. Higher scores reflect a more frequent report of the cognitions, emotions and behaviors included in the scale. Higher scores reflect a more frequent report of the cognitions, emotions and behaviors included in the scale. Bold-printed values indicate significant values at level p<.05 | | | |

| **Supplementary Table 3**  *Median scores and ranges of scores on the WSSCS-D and educational level* | | | | | | | | | | | | |
| --- | --- | --- | --- | --- | --- | --- | --- | --- | --- | --- | --- | --- |
|  |  | Control | | | | | |  | | DSD | | |
|  | *n* | | Agentic | | Negative | | Loyalty | *n* | Agentic | | Negative | Loyalty |
|  |  | | Sexuality | | Associations | |  |  | Sexuality | | Associations |  |
|  |  | | *Mdn (range)* | | *Mdn (range)* | | *Mdn (range)* |  | *Mdn (range)* | | *Mdn (range)* | *Mdn (range* |
| Primary school, lower secondary | 11 | 83 (63-94) | | 23 (16-44) | | 23 (15-28) | | 6 | 68.5 (15-98) | | 37.5 (22-53) | 19.5 (6-28) |
| Higher secondary | 143 | 72 (18-95) | | 30 (16-69) | | 22 (8-28) | | 55 | 65 (14-92) | | 35 (16-72) | 23 (6-28) |
| Higher education short type | 107 | 73 (21-98) | | 27 (16-85) | | 21 (6-28) | | 18 | 59.5 (48-78) | | 33 (21-81) | 21.5 (18-26) |
| Higher education long type | 319 | 73 (28-97) | | 28 (16-80) | | 22 (4-28) | | 8 | 63.5 (16-77) | | 33.5 (17-46) | 23 (8-26) |
| *p-value* |  | .256 | | .390 | | .155 | |  | | .971 | .701 | .634 |
| *Abbreviations. Mdn =* Median score; WSSCS-D = Women's Sexual Self-concept Scale-Dutch. | | | | | | | | | | | | |
| *Absolute scoring ranges.* Agentic Sexuality 14-98, Negative Associations 16-119, Loyalty 4-28. Higher scores reflect a more frequent report of the cognitions, emotions and behaviors included in the scale. Higher scores reflect a more frequent report of the cognitions, emotions and behaviors included in the scale. | | | | | | | | | | | | |

| **Supplementary Table 4**  *Mean scores and standard deviations on the BIS in DSD and control women aged 20-40 years* | | | |
| --- | --- | --- | --- |
|  | External genitals  *M (SD)* | Other sex-specific  characteristics *M (SD)* | Non sex-specific  characteristics  *M (SD)* |
| DSD (*n* = 60) | 8.47 (2.69) | 16.94 (4.19) | 54.13 (12.75) |
| Control (*n* = 70) | 6.83 (1.81) | 17.77 (3.40) | 52.05 (10.85) |
| *p-value* | ***<.001*** | *.23* | *.32* |
| *Abbreviations.* *M* = Mean score, *SD* = standard deviation.  *Absolute scoring ranges.* External genitals: 3-15 , Other sex-specific characteristics: 7-35, Non-sex-specific characteristics: 23-115. Higher scores reflect greater dissatisfaction. Bold-printed values indicate significant values at level p<.05 | | | |

| **Supplementary Table 5**  *Mean scores and standard deviations on the BIS in Caucasian DSD and control women* | | | |
| --- | --- | --- | --- |
|  | External genitals  *M (SD)* | Other sex-specific  characteristics *M (SD)* | Non sex-specific  characteristics  *M (SD)* |
| DSD (*n* = 78) | 8.55 (2.50) | 17.42 (3.96) | 54.07 (11.28) |
| Control (*n* = 63) | 6.92 (1.69) | 17.89 (3.11) | 53.25 (10.78) |
| *p-value* | ***<.001*** | *.440* | *.667* |
| *Abbreviations.* *M* = Mean score, *SD* = standard deviation.  *Absolute scoring ranges.* External genitals: 3-15 , Other sex-specific characteristics: 7-35, Non-sex-specific characteristics: 23-115. Higher scores reflect greater dissatisfaction. Bold-printed values indicate significant values at level p <.05 | | | |

| **Supplementary Table 6**  *Median scores and range of scores on the BIS and educational level* | | | | | | | | | | |
| --- | --- | --- | --- | --- | --- | --- | --- | --- | --- | --- |
|  |  | Control | | |  | DSD | | | | |
|  | *n* | External genitals  *Mdn (range)* | Other sex-specific charcteristics  *Mdn (range)* | Non sex-specific characteristics  *Mdn (range)* | *n* | External genitals  *Mdn (range)* | | Other sex-specific charcteristics  *Mdn (range)* | | Non sex-specific characteristics  *Mdn (range)* |
| Primary school, lower secondary | 3 | 9 (6-9) | 19 (18-21) | 50 (42-53) | 9 | 9 (3-14) | 17.5 (7-25) | | 52.5 (22-84) | |
| Higher secondary | - |  |  |  | 58 | 8.5 (4-15) | 17 (7-27) | | 55.5 (28-97) | |
| Higher education, short type | 9 | 6 (6-10) | 16 (15-23) | 53 (47-69) | 18 | 8 (6-15) | 16 (11-22) | | 51 (43-64) | |
| Higher education long type | 58 | 6 (3-11) | 18 (11-28) | 50(27-76) | 10 | 8 (5-12) | 18 (12-23) | | 54 (27-71) | |
| *p-value* |  | .517 | .654 | .418 |  | .939 | .876 | | .683 | |
| *Abbreviations. Mdn =* Median score.  *Absolute scoring ranges.* External genitals: 3-15 , Other sex-specific characteristics: 7-35, Non-sex-specific characteristics: 23-115. Higher scores reflect greater dissatisfaction. | | | | | | | | | | |
